# Characterising variability and predictors of infant mortality in urban settings: findings from 286 Latin American cities

**DOI:** 10.1136/jech-2020-215137

**Published:** 2020-10-14

**Authors:** Ana F Ortigoza, José A Tapia Granados, J Jaime Miranda, Marcio Alazraqui, Diana Higuera, Georgina Villamonte, Amélia Augusta de Lima Friche, Tonatiuh Barrientos Gutierrez, Ana V Diez Roux

**Affiliations:** 1 Urban Health Collaborative, Drexel University, Philadelphia, Pennsylvania, USA; 2 History and Political Science, Drexel University, Philadelphia, Pennsylvania, USA; 3 CRONICAS Centre of Excellence in Chronic Diseases, Universidad Peruana Cayetano Heredia, Lima, Peru; 4 Instituto de Salud Colectiva, Universidad Nacional de Lanus, Lanus, Argentina; 5 Escuela de Medicina, Universidad de Los Andes, Bogota, Colombia; 6 School of Medicine, Universidade Federal de Minas Gerais Faculdade de Medicina, Belo Horizonte, Brazil; 7 Instituto Nacional de Salud Publica, Mexico DF, Mexico

**Keywords:** Infant mortality, Urbanisation, Social inequalities, Public health policy

## Abstract

**Background:**

Urbanisation in Latin America (LA) is heterogeneous and could have varying implications for infant mortality (IM). Identifying city factors related to IM can help design policies that promote infant health in cities.

**Methods:**

We quantified variability in infant mortality rates (IMR) across cities and examined associations between urban characteristics and IMR in a cross-sectional design. We estimated IMR for the period 2014–2016 using vital registration for 286 cities above 100 000 people in eight countries. Using national censuses, we calculated population size, growth and three socioeconomic scores reflecting living conditions, service provision and population educational attainment. We included mass transit availability of bus rapid transit and subway. Using Poisson multilevel regression, we estimated the per cent difference in IMR for a one SD (1SD) difference in city-level predictors.

**Results:**

Of the 286 cities, 130 had <250 000 inhabitants and 5 had >5 million. Overall IMR was 11.2 deaths/1000 live births. 57% of the total IMR variability across cities was within countries. Higher population growth, better living conditions, better service provision and mass transit availability were associated with 6.0% (95% CI −8.3 to 3.7%), 14.1% (95% CI −18.6 to −9.2), 11.4% (95% CI −16.1 to −6.4) and 6.6% (95% CI −9.2 to −3.9) lower IMR, respectively. Greater population size was associated with higher IMR. No association was observed for population-level educational attainment in the overall sample.

**Conclusion:**

Improving living conditions, service provision and public transportation in cities may have a positive impact on reducing IMR in LA cities.

## BACKGROUND

Infant mortality (IM) is often considered a marker of the degree of a country’s social and economic development because infant wellness and survival are closely related to the conditions into which infants are born and in which they grow.^1^ In low- and middle-income countries, infant mortality rates (IMR) have dropped significantly over last three decades, mostly due to reductions in deaths caused by conditions preventable or treatable through sanitation, maternal and perinatal care, and immunisation coverage.^2^ By 2010, LA showed the lowest IMR among developing regions, although the rate of decline has experienced a slowdown compared to other regions since 2005.^3^ This stagnation in the decrease of mortality rates could result from the fact that once easily preventable causes of death are tackled, achieving further reductions requires addressing drivers of mortality related to social inequalities in the population.

LA is one of the most urbanised regions in the world.^4^ While cities have been seen as places of economic opportunities and better access to services such as education and healthcare,^5^ many aspects of life in cities can negatively affect infant and child health. The accelerated urban population growth that occurred over the last three decades resulted in rapid expansion of many cities with inadequate urban planning.^6^ Cities can have hazardous levels of air pollution,^7^ which has been linked to premature birth, and higher morbidity and mortality among infants.^8 9^ In addition, almost 20% of the population currently lives in poverty.^6 10^ This creates a particularly vulnerable environment for infants as a result not only of poorer access to care but also because of greater exposure to social and environmental conditions hazardous to health.^10^ This heterogeneity in urban environment observed within and across urban areas in LA challenges the idea of ‘urban advantage’, by which cities are believed to have lower maternal and IMR compared to rural areas.^11^

Social inequalities linked to child mortality have been extensively described at the national or subnational levels^12 13^; however, analyses at the city level have been mostly restricted to a few big metropolitan areas.^14 15^ Examining how characteristics of cities affect mortality and health outcomes is important to identify actions and policies to improve infant health and promote health equity in the context of a rapidly urbanising world. We used data from a unique multinational urban health collaboration to quantify differences in IMR across a wide range of cities in LA and to examine how key features of cities are related to IMR.

## METHODS

### Sample

Data for this study are drawn from the SALURBAL project (SALud URBana en America Latina- Urban Health in Latin America), which includes all cities of 100 000 or more inhabitants in 2010 in 11 countries for a total of 371 cities. Each city was defined geographically by administrative units (ie, municipios, comunas, partidos, delegaciones, cantones or corregimientos) that encompassed the urban extent of the city in 2010 using satellite imagery.^16^ For this study, we included cities for which vital statistics registries were available from 2014 to 2016 and presented good quality of death registry based on a separate analysis of adult mortality.^17^ We assumed that cities with good levels of registration for adult deaths (coverage of 90% or above) also have good coverage of deaths among infants. We included 286 cities in Argentina, Brazil, Chile, Colombia, Costa Rica, Mexico, Peru and Panama. Eleven cities (five cities in Nicaragua, three cities in Guatemala and El Salvador, respectively) were excluded due to lack of vital registration for the years of study. A total of 74 cities (9 cities in Brazil, 19 in Colombia, 31 in Mexico and 15 in Peru) were excluded because the estimated coverage of adult mortality was considered of low quality. Mean level of mortality coverage in all excluded cities was below 85% and excluded cities had poorer living conditions and lower provision of water connected to public network compared to cities included in this study (online supplemental material).

10.1136/jech-2020-215137.supp1 Supplementary data

### Outcome

*IMR*. We calculated IMR (deaths less than 1 year of age per 1000 live births) for the period 2014–2016. Deaths and live birth were retrieved based on deceased’s and maternal place of residence, respectively. Three years were pooled to increase the stability of the estimates.

### Exposures

We explored several characteristics of cities that we hypothesised would be related to IMR based on prior literature.^6 10 13^

*City population:* we obtained population data from national censuses that provided estimates or projections for years 2010 and 2015. We investigated population size in 2010 as a continuous variable and categorised as (1) below a quarter million, (2) greater or equal to quarter million to less than half million, (3) greater or equal to half million to less than one million, (4) greater or equal to one million to less than five million, and (5) greater or equal to five million.

*Annual population growth rate (APGR):* this was calculated based on differences in population size between 2015 and 2010.

*Urban* s*ocial* e*nvironment*: we retrieved census indicators and harmonised them across countries for 12 socioeconomic (SE) variables describing education, housing, water, sanitation and employment. Using principal component analyses, we identified three components that incorporated seven variables. Variables related to employment (per cent of unemployed and per cent of labour force participation) and housing materials (per cent of houses with either durable walls or masonry walls) did not load onto any of the three components.

Each indicator was standardised to a mean of 0 and SD of 1, and indicators that loaded onto each component were summed to create three SE scores. Each score may be related to IMR through different mechanisms:

- Score of living conditions (related to housing and poverty), including (1) per cent of households with piped water inside the dwelling; (2) per cent of households with overcrowding (more than three people per room, excluding kitchen and bathroom); and (3) per cent of population aged 15–17 years attending school, as a marker of social inclusion, since low school attendance among adolescents has been linked to poverty^18^ and exclusion from productive systems.^19^ We reverse coded the overcrowding indicator so that higher score values signify better living conditions.
- Score of service provision (related to public services that cities provide to dwellings), including (1) per cent of households with access to water from a municipal public or private water network, and (2) per cent of households with sewage system connected to a municipal public or private sewage network. Higher score values signify better service provision.
- Score of educational attainment (as a marker of SE context) including (1) per cent of population aged 25 years or above that has completed high school level or above, and (2) per cent of population aged 25 years or above that completed university level or above. This score characterises SE aspects of cities different from living conditions and service provision captured in the other two scores.^20^ Higher score values signify better educational achievement in the population.

*Urban built environment*: we created an indicator of mass transit availability based on the presence of subway and bus rapid transit (BRT) networks in the city. Mass transit availability was considered as present if the city had either subway or BRT network available, and absent when none of these options were present.

### Other variables

*Healthcare access*: access to healthcare services for infants was proxied by the city coverage of first dose of triple viral vaccine (MMR1, measles–mumps–rubella vaccine) among the population of 1-year-olds. MMR1 presents a schedule that is similar across countries making it suitable for harmonisation. Data for the year 2016 were provided by WHO.^21^

*National gross domestic product per capita* (GDP per capita): we retrieved the national real GDP (output-based) per population for 2015 for each country from Penn World Tables^22^ and use the median value of the sample (US$15 530.7) to categorise countries in two groups: above and below the median GDP per capita. We stratified on GDP because country economic conditions could modify the importance of the city-level factors, we studied for IM.

### Statistical analysis

We described the distribution of city characteristics by categories of city population size. We examined the distribution of IMR and city-level predictors in the overall sample and by countries. Since IMR was approximately normally distributed in our sample, we decomposed the total variance in IMR across and within countries using a linear mixed model with a random intercept for each country and no covariates. We then estimated the association of city-level variables with IMR using a Poisson multilevel model. City-level predictors were first explored separately and then combined. To determine whether associations of city variables with IMR were independent of access to healthcare, we further adjusted for MMR1 coverage.

We explored effect modification by country GDP per capita by repeating analyses in countries with GDP per capita above and below the median value of the sample, and by testing for statistical interactions between GDP per capita and city-level predictors.

## RESULTS

Selected characteristics of the cities are shown in table 1. Of the 286 cities, about half (45.5%) had less than a quarter million inhabitants and only five had over five million. Mean annual growth rates over the past 5 years were positive in all size categories and were slightly lower in the largest cities (4.2% in cities of five million or more compared to around 7% per year for other cities). SE indicators did not differ substantially across city size. Cities over one million tended to have higher proportions of households with piped water in the dwelling than cities with less than one million, but differences across city sizes were not statistically significant (table 1). Cities below a quarter million showed lower mean levels of education: 36.4% of the population with complete high-school or above, and 10.3% of population with complete university or above compared to about 42% and 14% in the rest of the cities, respectively. Cities below a quarter million did not have subways or BRT, and availability of mass transit was higher in larger cities: while only 25.5% and 4.3% of the cities between half to one million residents had BRT and subway systems, respectively, all cities with more than five million residents had both BRT and subways. The mean level of vaccine coverage in the sample was 91.3% and did not differ by city size. Mean IMR was 11.2 deaths per 1000 live births and only cities over five million had IMR below the mean. Cities in Argentina, Chile, Colombia and Costa Rica showed mean IMR below the overall mean for the full sample (table 1).

**Table 1:** Demographic and socioeconomic characteristics of cities by city population size (N=286 cities)

	Total	City population size*					
	n (% distribution) or mean (SD)	100 000–250 000	250 000–500 000	500 000–1 Million	1–5 Million	>5 Million	P value
Overall number of cities, n (%)	286	130 (45.5%)	70 (24.5%)	47 (16.4%)	34 (11.9%)	5 (1.7%)	
Number of cities by country, n (col%)							
Argentina	33 (11.5%)	15 (11.5%)	8 (11.4%)	5 (10.6%)	4 (11.8%)	1 (20.0%)	
Brazil	143 (50.4%)	74 (56.9%)	31(44.3%)	19 (40.4%)	17 (50.0%)	2 (40.0%)	
Chile	21 (7.3%)	12 (9.3%)	6 (8.5%)	2 (4.3%)	–	1 (20.0%)	
Colombia	16 (5.6%)	6 (4.7%)	5 (7.0%)	4 (8.5%)	1 (2.9%)	–	
Costa Rica	1 (0.3%)	–	–	–	1 (2.9%)	–	
Mexico	61 (21.3%)	19 (14.6%)	16 (22.9%)	15 (31.9%)	10 (29.4%)	1 (20.0%)	
Panama	3 (1.0%)	2 (1.6%)	–	–	1 (2.9%)	–	
Peru	8 (2.8%)	2 (1.6%)	4 (5.6%)	2 (4.3%)	–	–	
Population growth rate†, mean% (SD)	6.5 (3.3)	6.5 (3.7)	6.7 (3.2)	6.6 (2.5)	6.4 (2.6)	4.2 (1.1)	0.559
1. Score of Living conditions, mean (SD)	0.06 (0.7)	0.13 (0.7)	−0.02 (0.7)	−0.03 (0.7)	0.08 (0.4)	0.22 (0.4)	0.613
% of households with piped water in the dwelling	89.9 (11.4)	91.1 (11.1)	87.5 (13.3)	88.7 (11.8)	91.7 (7.2)	92.6 (7.2)	0.227
% of households with overcrowding‡	5.0 (4.0)	4.5 (4.3)	5.5 (4.0)	5.6 (3.9)	4.8 (3.0)	5.2 (2.6)	0.429
% of population 15–17 attending school	80.3 (7.6)	80.7 (7.9)	80.2 (7.6)	79.5 (6.9)	79.4 (7.5)	83.4 (5.0)	0.691
2. Score of Service provision, mean (SD)	−0.08 (0.4)	−0.1 (0.6)	−0.08 (0.4)	−0.03 (0.4)	−0.07 (0.4)	0.12 (0.4)	0.766
% of households with water connected to municipal network	90.8 (10.6)	90.3 (10.9)	90.1 (11.9)	91.7 (9.0)	91.89 (8.9)	92.1 (9.1)	0.822
% of households with sewage system connected to municipal network	70.0 (25.2)	68.9 (26.8)	70.5 (24.3)	72.3 (24.3)	68.2 (22.9)	83.7 (16.2)	0.681
3. Score of Population Educational attainment, mean (SD)	−0.22 (0.3)	−0.35 (0.3)	−0.19 (0.3)	−0.04 (0.3)	−0.004 (0.3)	−0.03 (0.2)	<0.0001
% Population 25+ with complete high school or above	38.8 (7.7)	36.4 (7.4)	39.7 (8.1)	41.5 (6.9)	41.6 (6.8)	43.5 (3.7)	<0.0001
% Population 25+ with complete university level or more	12.2 (4.2)	10.7 (3.8)	12.2 (4.1)	14.3 (4.0)	14.9 (3.4)	13.6 (3.9)	<0.0001
4. Availability of mass transit, n (col%)	41 (14.3%)	–	3 (4.2%)	13 (27.7%)	20 (58.8%)	5 (100%)	<0.0001
Bus rapid transit	40 (14.0%)	–	3 (4.2%)	12 (25.5%)	20 (58.8%)	5 (100%)	<0.0001
Subway	16 (5.6%)	–	–	2 (4.3%)	9 (26.5%)	5 (100%)	<0.0001
MMR1 coverage, mean % (SD)	91.3 (13.8)	91.1 (13.2)	90.2 (15.3)	93.1 (12.2)	92.0 (14.9)	91.6 (9.24)	0.855
Overall infant mortality rate¶, mean (SD)	11.2 (2.8)	11.1 (2.9)	11.4 (2.8)	11.1 (2.6)	11.4 (2.3)	10.8 (2.5)	0.877
Argentina	10.0 (2.2)	9.5 (1.9)	11.3 (3.0)	9.4 (1.7)	9.5 (1.7)	10.0 (.)	0.2976
Brazil	11.9 (2.4)	11.7 (2.4)	11.9 (2.1)	11.9 (3.1)	12.4 (2.3)	12.1 (1.2)	0.8537
Chile	7.4 (0.9)	7.6 (1.1)	7.2 (0.6)	6.5 (0.3)	–	6.8 (.)	0.3523
Colombia	10.8 (2.8)	9.8 (2.3)	12.9 (3.0)	10.3(2.0)	7.9 (.)	–	0.1735
Costa Rica	7.5(.)	–	–	–	7.5 (.)	–	–
Mexico	11.8 (2.4)	12.7 (3.2)	11.0 (1.5)	11.6 (1.87)	11.4 (1.9)	13.0 (.)	0.3445
Panama	13.1 (2.2)	14.2 (1.7)		–	11.0 (.)	–	0.3573
Peru	11.2 (4.7)	7.04 (3.0)	13.5 (5.6)	11.0 (1.21)	–	–	0.3414

*City population size is defined by the population in 2010.

†Population growth for the period 2010–2015.

‡Overcrowding is defined as more than three people per room, excluding kitchen and bathroom in a household.

§Mass transit availability refers to the presence of either subway or bus rapid transit networks.

¶Infant mortality rate=number of infant deaths per 1000 live births.

MMR1, measles–mumps–rubella vaccine.

**Figure 1:**
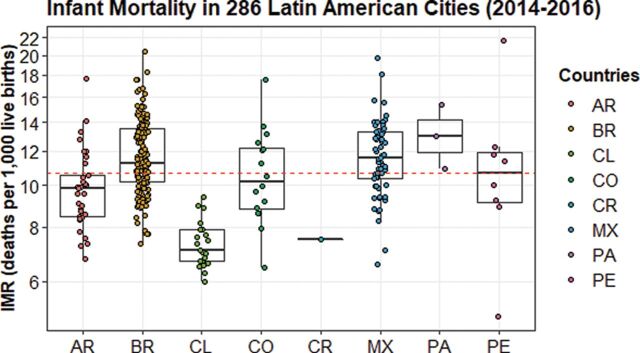
Distribution of infant mortality rate (IMR) in cities by country, 2014–2016 (n=286 cities). Each dot represents a city-estimate of IMR, and boxplots show the country distribution of IMR. Dashed line describes the median IMR of the overall sample (11 deaths per 1000 live births).

AR, Argentina; BR, Brazil, CL, Chile; CO, Colombia; CR, Costa Rica; MX, Mexico; PA, Panama; PE, Peru.

Figure 1 shows distribution of IMR for cities within countries. Although median IMR differed across countries, the greatest heterogeneity is observed across cities within countries. The intraclass correlation was 0.43 implying that 57% of the total variability in city-level IMR was within countries.

Table 2 shows estimated per cent differences in IMR associated with a 1SD higher population size, APGR, SE scores, mass transit availability and 1% higher MMR1 coverage. In the models with each exposure separately, the three SE scores showed negative associations with IMR. When all exposures were adjusted for each other, higher city growth, better living conditions, better service provision and availability of mass transit were associated with lower IMR: 1SD higher APGR was associated with 6.0% lower IMR (95% CI −8.3 to −3.7%), 1SD higher score of living condition with 14.1% lower IMR (95% CI −18.6 to −9.2), 1SD higher score of service provision with 11.4% lower IMR (95% CI −16.1 to −6.4), and the presence of mass transit with 6.6% lower IMR (95% CI −9.2 to −3.9). No association was observed for population-level educational attainment in the multivariable model. One SD higher population size was associated with 0.7% higher IMR (95% CI 0.1 to 1.4). These results did not change substantially after accounting for MMR1 coverage.

**Table 2:** Estimated per cent differences in IMR associated with city-level predictors (n=286)

	Single-exposure models		Multivariable‡‡		Adjusted for healthcare§§	
	% Difference	95% CI	% Difference	95% CI	% Difference	95% CI
Population size*	−0.1	−0.9 to 0.8	0.7	0.1 to 1.4	0.8	0.2 to 1.3
Population growth rate†	−3.6	−6.8 to −0.3	−6.0	−8.3 to −3.7	−6.0	−8.3 to −3.6
Living conditions score‡	−18.4	−26.7 to −9.2	−14.1	−18.6 to −9.2	−14.1	−18.8 to −9.2
Services provision score§	−13.2	−17.1 to −9.2	−11.4	−16.1 to −6.4	−11.0	−15.5 to −6.2
Population educational attainment score¶	−13.7	−21.7 to −4.8	1.4	−1.2 to 4.1	0.9	−1.7 to 3.6
Availability of mass transit**	−3.9	−11.2 to 4.0	−6.6	−9.2 to −3.9	−6.7	−9.2 to −4.2
MMR1 coverage††					−0.1	−0.2 to 0.04

*Population size is defined as 1SD higher the population in 2010.

†Population growth rate is defined as 1SD higher annual difference in population size from 2010 to 2015.

‡Living conditions score includes per cent of households with piped water in the dwelling, per cent of households with overcrowding (3+ per room) in the house and per cent of population aged 15–17 years attending school;

§Services provision score includes per cent of households with water connected to municipal network and per cent of households with sewage system connected to municipal network.

¶Population-level educational attainment score includes per cent of population aged 25 years or above with complete high school level or above, per cent of population aged 25 years or above with complete university level or above.

**Mass transit availability refers to the presence of either subway or bus rapid transit networks.

††MMR1 coverage represents the percentage of 1-year-old children, who received the first dose of MMR1 among overall population of 1-year-olds.

‡‡Multivariable model includes all city-level predictors except MMR1 coverage.

§§Includes all variables in multivariable model and MMR1 coverage.

Estimates correspond to per cent differences in IMR for 1SD higher SE scores, population size and growth, 1% higher MMR1 coverage and the availability of mass transit.

IMR, infant mortality rate; MMR1, first dose measles–mumps–rubella vaccine; SE, socioeconomic; 1SD, one SD.

When countries were grouped by GDP per capita (table 3), better living conditions and mass transit availability were similarly associated with lower IMR at high and low GDP levels, but better service provision was associated with lower IMR only for countries of lower GDP (p value for interaction 0.0001). Higher population growth was also similarly associated with lower IMR at higher and lower levels of GDP, but greater population size appeared to be more strongly associated with higher IMR in countries with higher GDP (p value for interaction 0.002). Although a statistically significant interaction with country GDP was also observed for education, CIs were very wide in both GDP strata.

**Table 3:** Estimated percentage difference in IMR associated with city-level predictors stratified by country GDP per capita

	Countries below median GDP‡‡ n=168 cities		Countries above median GDP§§ n=118 cities		Test for interaction¶¶
	% Difference	95% CI	% Difference	95% CI	P value
Population size*	0.4	−0.2 to 0.9	1.2	−0.5 to 3.0	0.0017
Population growth rate†	−6.5	−9.8 to −3.1	−5.9	−9.8 to −1.9	0.3833
Living conditions score‡	−18.0	−26.7 to −8.2	−16.3	−19.0 to −13.5	0.0469
Services provision score§	−11.7	−14.4 to −8.8	2.7	−0.7 to 6.3	<0.0001
Population educational attainment score¶	0.1	−2.0 to 2.1	4.9	−6.3 to 17.4	0.0067
Availability of mass transit*	−6.6	−9.9 to −3.2	−7.5	−18.1 to 4.5	0.1784
MMR1 coverage††	−0.2	−0.2 to −0.1	0.01	−0.4 to 0.5	0.2766

*Population size is defined as 1SD higher the population in 2010.

†Population growth rate is defined as 1SD higher annual difference in population size from 2010 to 2015.

‡Living conditions score includes per cent of households with piped water in the dwelling, per cent of households with overcrowding (3+ per room) in the house and per cent of population aged 15–17 years attending school.

§Services provision score includes per cent of households with water connected to municipal network and per cent of households with sewage system connected to municipal network.

¶Population-level educational attainment score of 3 includes per cent of population aged 25 years or above with complete high school level or above and per cent of population aged 25 years or above with complete university level or above.

**Mass transit availability refers to the presence of either subway or bus rapid transit networks;

††MMR1 coverage represents the percentage of 1-year-old children who received the first dose of measles-mumps-rubella vaccine among overall population of 1-year-olds.

‡‡Countries with GDP/capita below the median (US$15 530): Brazil, Colombia, Costa Rica and Peru.

§§Countries with GDP/capita above the median (US$15 530): Argentina, Chile, Mexico and Panama.

¶¶Each interaction was tested separately along with the full model. GDP was coded as GDP=0 for countries with GDP/capita below the median, and GDP=1 for countries above the median.

Estimates correspond to per cent differences in IMR for 1SD higher SE scores, population size and growth, 1% higher MMR1 coverage and the availability of mass transit.

GDP, gross domestic product; IMR, infant mortality rate; MMR1, measles–mumps–rubella vaccine; SE, socioeconomic; 1SD, one SD.

To explore the influence of outliers, we carried out sensitivity analysis excluding cities above the 99th or below the 1st percentile of IM and found similar results. The exclusion of Peru (which has been reported to have significant undercounting of infant deaths in some studies^23^) also yielded similar results.

## DISCUSSION

This study showed substantial heterogeneity in IMR across LA cities. Although countries differed in IMR, most of the variability in city-level IMR (almost 57%) was within countries. Higher population growth rate, better living conditions and services provision and availability of mass transit were associated with lower IMR, independently of access to healthcare. Greater population size was associated with higher IMR. GDP per capita modified these associations, such that services provision was associated with lower IMR only in countries with GDP per capita below the median.

The large heterogeneity in IMR observed across cities (even within the same country) highlights the importance of examining city-level factors as predictors of IMR. In our study, city-level measures of living conditions (reflecting housing, crowding and schooling in adolescents) and services provision (water and sanitation) were independently associated with lower IMR. Quality housing as well as access to water and sanitation services are known to reduce the transmission of respiratory and enteric diseases,^24^ which remain the main causes of death among infants.^25^ Prior work has shown that urban slum prevalence is related to IMR across countries,^26 27^ but these studies did not identify the features of slums that may be most important and ignore heterogeneities across cities. We show that specific living conditions and service provision at the city level are related to IMR in cities, highlighting the potential role of city-level policies affecting these factors in reducing IMR in urban areas.

We also found that higher population growth was associated with lower IMR while greater population size was related to higher IMR, after accounting for other city predictors. Further analyses are needed to examine how the population dynamics of cities may affect IMR. Population growth could reflect migration and/or natality rates. Decreased fertility and natality associated with improved status of women have been linked to lower IMR.^28^ Increased population growth at the expense of migration from rural areas may reflect the influx of disadvantaged populations and may create demands that cities are unable to face resulting in higher IMR.^29^ However, we found that greater growth was associated with lower IMR suggesting that growth may be proxying other unmeasured factors related to lower IMR.

A novel aspect of our study was the investigation of public transportation, which has been hypothesised to impact health.^30^ We found that availability of BRTs and subways was associated with lower IMR independently of city population size and other city-level predictors. Availability of timely, rapid and extensive public transportation network may be key to reduce inequalities in access to healthcare related to IMR, as well as in access to employment opportunities and consequently better SE position for dwellers living in impoverished city areas. To our knowledge, this is the first study exploring the relationship between city transport and IMR.^30^ Given limitations in the information, we used to characterise public transportation these preliminary findings deserve further investigation.

Country economic development appeared to modify some of the associations between city-predictors and IMR. We found that better service provision was only associated with lower IMR in cities of countries with GDP per capita below the median. This may be related to greater variability in these predictors across cities in countries with lower GDP income or to the fact they are relevant predictors in these contexts because of the causes of death driving IMR.^2^

Our study is unique and unprecedented in that we have compiled and harmonised data on urban environment, and IMR across nearly 300 cities in LA. We were able to describe heterogeneity in IMR across cities and study how a range of city-level factors are related to IMR. Although many studies have focused on IMR in urban areas, to our knowledge, this is the first investigation examining the influence of city social and built environments on IMR across multiple cities. Although several previous studies have documented associations of social and economic factors with IMR at the country level,^31 32^ few if any studies have examined these factors at the level of cities. The study of city-level factors is especially relevant to the development of local interventions. A growing body of work has documented how local interventions in cities can improve infant health.^33^ For example, housing improvements and water and sanitation provision,^34 35^ or more comprehensive urban redevelopment interventions^36^ have been linked to reduction in respiratory and enteric infections^34 35^ as well as in IM^36^ in urban slums in LA.

Because of the number of cities studied, some of the measures available had limitations. Measures of public transportation were limited to subways and BRTs. These have advantages to traditional buses, but they do not fully capture transit availability. We did not include other potentially relevant physical environment features such as air pollution or density of green areas. The characterisation of healthcare access for infants was restricted to coverage of MMR1, a single-dose vaccine that tends to be more equally distributed in the population than interventions requiring specialised personnel or complex technology.^37^ This measure clearly has important limitations and does not adequately capture the complexity of healthcare access. A better representation of healthcare access may require the combination of several indicators related to family planning, antenatal care, multiple vaccine coverages and access to treatment during disease episodes like diarrhoea or pneumonia.^37^ Lastly, our city-level analyses, although informative, ignore heterogeneity across neighbourhoods within cities.

Finally, we restricted our study to cities with good quality of mortality registries based on adult mortality estimations with the purpose of generating unbiased estimates of IM. However, it is possible that registration of infant deaths and live births could be conditioned by other determinants of under-registration different from the ones related to adult mortality. There is evidence that undercounting of child deaths may be especially problematic in some areas of Peru^23^ but sensitivity analyses excluding Peru resulted in similar findings. If more under-registration of infant deaths is associated with lower SE conditions, the associations we report may be underestimates of true associations. Future studies involving IM need to address under-registration through specific approaches that estimate IMR, like indirect demographic methods or statistical methods designed for small-area estimations.

## CONCLUSION

In an increasingly urbanised world, it is critical to identify which urban policies are necessary to improve population health. Our city-level analyses show that features of urban social and physical environments are related to important differences in IMR across cities. Prioritisation of urban policies and interventions related to improving living conditions, sanitary services and public transportation availability may be necessary to have positive and sustained impacts on infant survival in urban areas.

**What is already known on this subject**

- Although many studies have described differences in infant mortality across countries or regions, little research has explored heterogeneity across cities within countries. This heterogeneity may be especially important in nations where rapid urbanisation has been heterogeneous. Identifying which aspects of the urban environment contribute to differences in infant mortality is important to inform policies and programmes to improve infant health.

**What this study adds**

- We found that in Latin America, although infant mortality rates (IMR) differ across countries, large heterogeneity is observed across cities within countries. Higher IMR were observed in cities with higher proportions of residents experiencing social exclusion, inadequate housing conditions and insufficient municipal provision of sanitary services. Availability of mass transit in cities was associated with lower IMR.
- Achieving further reductions in infant mortality in Latin America will require focusing on reducing social inequities in urban populations and prioritising interventions related to improving living conditions, sanitary services and public transportation.
