# Supplementary material for: Characterising variability and predictors of infant mortality in urban settings: findings from 286 Latin American cities
Source: J Epidemiol Community Health. 2020 Oct 14;75(3):264–70. doi: 10.1136/jech-2020-215137 (PMC7892385; doi:10.1136/jech-2020-215137)
Supplement: Supplementary data [file jech-2020-215137supp001.pdf]

Supplementary material.

Table S1. Characteristics included vs excluded cities

	Total n (% of sample) or mean (SD)	Included in the sample	Excluded from the sample	p-value
Overall number of cities, n (%)	360	286 (79·4%)	74 (20·5%)	
Population growth rate [a], mean% (SD)	6·8 (4·0)	6·9 (3·7)	6·6 (5·1)	0·635
Socio-economic predictors, mean (SD)				
1.Poverty and living conditions				
% of households with piped water in the dwelling	87·1 (13·3)	89·7 (11·5)	77·0 (15·2)	<0·001
% of households with overcrowding in the house [b]	5·8 (4·6)	4·9 (3·9)	9·2 (5·1)	<0·001
% of population 15-17 attending school	79·1 (7·9)	80·4 (7·5)	74·6 (7·7)	<0·001
Score	0·002(0·7)	0·14 (0·6)	-0·51(0·6)	<0·001
2.Service provision				
% of households with water connected to municipal network	88·8 (12·0)	90·0 (10·5)	84·4 (15·7)	<0·001
% of households with sewage system connected to municipal network	70·2 (24·4)	69·9 (25·2)	70·6 (21·5)	0·836
Score	0·002 (0·4)	0·03(0·4)	-0·08(0·4)	0·047
3.Educational attainment				
% population 25+ with complete high school or above	38·9 (9·0)	38·6 (7·7)	39·4(13·1)	0·502
% population 25+ with complete university level or more	12·3 (4·4)	12·1 (4·2)	13·1 (5·1)	0·097
Score	0·002(0·4)	-0·01 (0·3)	0·06 (0·6)	0·190
Availability of mass transit, n (% col)				
Bus rapid transit	48 (13·3%)	40 (14·0%)	8 (10·8%)	<0·001
Subway	18 (5·0%)	16 (5·6%)	2 (2·7%)	<0·001
Mass transit availability	49 (13·6%)	41 (14·3%)	8 (10·8%)	<0·001
MMR1 coverage mean % (SD)	11·3 (3·1)	11·2 (2·7)	11·7 (4·3)	0·320
Overall Infant mortality rate [d] mean (SD)	90·9 (14·2)	91·2 (13·7)	89·5 (15·8)	0·282

SD = Standard deviation

(a) Population growth for the period 2010- 2015

(b) Overcrowding is defined as more than three people per room, excluding kitchen and bathroom in a household

(c) Mass transit availability refers to the presence of either subway or bus rapid transit (BRT) networks

(d) Infant Mortality rate = number of infant deaths per 1000 live births
